# Immunohistochemical Study of Progesterone Receptor Expression in Pleomorphic Adenoma and Mucoepidermoid Carcinoma of the Salivary Gland 

**DOI:** 10.30476/dentjods.2024.102344.2356

**Published:** 2025-09-01

**Authors:** Najmeh Jafari, Seyed Mostafa Mahmoudi, Mahmood Akhavan Tafti, Farinaz Sabaghzadegan

**Affiliations:** 1 Dept. of Oral and Maxillofacial Pathology, School of Dentistry, Shahid Sadoughi University of Medical Sciences, Yazd, Iran.; 2 Dept. of Oral and Maxillofacial Pathology, School of Dentistry, Social Determinant of Oral Health Research Center, Shahid Sadoughi University of Medical Sciences, Yazd, Iran.; 3 Dept. of Pathology, School of Medicine, Shahid Sadoughi University of Medical Sciences, Yazd, Iran.; 4 Dept. of Oral and Maxillofacial Pathology, School of Dentistry, Shahid Sadoughi University of Medical Sciences, Yazd, Iran.

**Keywords:** Progesterone, Immunohistochemistry, Pleomorphic adenoma, Mucoepidermoid carcinoma

## Abstract

**Background:**

Progesterone (PR) plays a role in the differentiation and growth of various tissues. One of the most common carcinogenic mechanisms of PR is increasing cell proliferation and inhibiting their apoptosis. There are contradictory results from various studies about the expression level of PR receptors in salivary tumors.

**Purpose:**

Considering the sporadic studies and the contradictory results, this study was conducted to determine the expression level of PR receptor in the most common benign and malignant salivary gland tumors.

**Materials and Method:**

In this descriptive cross-sectional study, 58 paraffinized blocks (36 pleomorphic adenoma samples and 22 mucoepidermoid carcinoma samples) were selected. PR receptor immunohistochemical staining was performed on the samples and the resulting slides were examined under a light microscope with 100x magnification. The gleaned data were analyzed with SPSS25 using descriptive statistics, Fisher’s exact test, and Chi-square test.

**Results:**

Out of 36 pleomorphic adenoma samples, one sample expressed PR receptor moderately and two expressed it weakly. Besides, out of 22 mucoepidermoid carcinoma samples, only one sample expressed PR receptor. The comparison of PR receptor expression between two groups was not statistically significant (*p* Value=0.719).

**Conclusion:**

Based on the findings of this study, it can be concluded that PR receptor cannot probably play a direct role in carcinogenesis and prognosis of benign and malignant salivary tumors.

## Introduction

Benign and malignant salivary gland tumors constitute a significant part of head and neck tumors second to oral squamous cell carcinoma. Mucoepidermoid carcinoma (MEC) is the most common malignant and pleomorphic adenoma (PA) is the most common benign salivary tumor [ 1 - 4 ]. Understanding the biology of salivary gland tumors is very important in their diagnosis, treatment, and prognosis. Steroid hormones regulate the growth, differentiation, and function of cells [ 5 - 6 ]. Estrogen and progesterone (PR) are two of the most common steroid hormones responsible for biological processes with the potential for anatomical and physiological changes in human development [ 1 ]. The importance of sex hormone receptors, such as PR, in breast cancer has been proven, and their role in determining prognosis and hormone therapy has been discussed . The similarity between breast tissue and salivary glands, in terms of the presence of acini structures, ducts and the simultaneous occurrence of breast and salivary gland carcinoma and higher incidence in women, suggests the possible role of these receptors in salivary gland tumors .Until now, surgery with or without chemotherapy and radiotherapy has sometimes been associated with local recurrence and metastasis [ 11 ]. The use of new therapeutic strategies based on biological drugs should be considered for disease control [ 11 ], since PR antagonists, such as mifepristone, as an adjunctive treatment in breast cancer patients increase survival and reduce cancer recurrence [ 12 ]. Some of the previous studies [ 13 - 17 ] have examined the expression of estrogen and PR receptors in some salivary gland tumors, and contradictory results have been achieved. Shick *et al*. [ 18 ] and Ozono *et al*. [ 19 ] found high expression of PR receptor in salivary tumors, while the level of PR expression was not high in the study by Kolude *et al*. [ 20 ]. In Kolude *et al*. study [ 20 ], PR receptor was expressed only in two cases of adenoid cystic carcinoma and one case of myoepithelioma, and it was not expressed in any of the samples of MEC and PA. Concerning the sporadic studies and the contradictory results yielded by these studies, this study was conducted to examine the expression level of PR receptor in the most common benign and malignant salivary gland tumors. 

## Materials and Method

This descriptive cross-sectional study was conducted in 2023 at Shahid Sadoughi School of Dentistry, Yazd, in collaboration with the Pathology Laboratory of Shahid Sadoughi Hospital, Yazd. In so doing, paraffinized blocks pertaining to PA and MEC were requested from the archive after examining the patient files. By using the appropriate formula, $n_{0}=\frac{Z^{2}p(1-p)}{e^{2}}$ the sample size was selected. A total of 58 tissue blocks, including 36 PA and 22 MEC samples with sufficient tissue, were selected. After recording the clinical information of the patients, their paraffin-embedded blocks were obtained for immunohistochemical procedures. Sections of 2-µm were then prepared from the paraffin blocks and that subsequently underwent dehydration and deparaffinization. Next, the sections were immersed in Tris Buffered Saline (TBS) buffer (Diagnostic Biosystem) with pH= 7.4 for 10min; then, to prevent non-specific staining, H2O2 blocker (DBS, America) was used in a dark environment by 1cc in 9cc of methanol for 10 min. In the next step, sections were washed with water, and recycling buffer with pH=9 was used in laboratory for refolding of proteins and separation of recombinant proteins. Subsequently, the sections were placed inside the microwave with maximum boiling power (900 watt) for 40 min and then, with one third power for 20 min. After cooling the sections, a hydrophobic pen (DAKP PEN) was used to enclose the tissue. At this stage, antibody was added on the sections against PR (Clone SP42, DBS, America) and washed with water (first with tap water and then with distilled water) after one hour; then it was immersed in TBS buffer with pH=7.4 for 10 min. Next, Enhancer (DBS, America) was added for 20 min and after that, polymer was added for 30 min. It was washed again with buffer with pH=7.4 and then 1 cc of substrate chromogen (DBS, America) solution was used. After washing with TBS and then with water, hematoxylin was added for 15min, and then the steps of immersion in xylene and alcohol were performed. The prepared slides were observed under a light microscope by two pathologists and the percentage of stained nuclei was determined with the formula 100x (number of thousands of tumoral cell nuclei in 10 random fields/number of positive nuclei). To assess PR expression, staining was classified as follows: <5%=negative, 5-20%= weak, 21-50%= moderate, and > 50%= strong [ 21 ]. 

To ensure the accuracy of the immunohistochemical staining technique used in this study, a positive and negative control sample was used. The positive control included a sample of breast cancer in which the nuclei of the cells were stained, and the negative control included a sample of breast cancer in which none of the nuclei were stained by removing the secondary antibody. 

## Results

Out of all the examined samples, the majority were found in the age range of 40+ years and in the major salivary glands. The majority of MEC cases were observed in males, while the majority of PA cases were seen in females. Most MEC had a low microscopic grade (54.54%) (Table 1). 

**Table 1:** Frequency of pleomorphic adenoma and mucoepidermoid carcinoma in terms of background variables

Tumor type	Background variable		f	%
PA	Age	<40	14	38.88
		≥40	22	61.12
	Gender	Male	14	38.88
		Female	22	61.12
	Involved location	Major salivary glands	27	75
		Minor salivary glands	9	25
MEC	Age	<40	9	40.9
		≥40	13	59.1
	Gender	Male	12	54.54
		Female	10	45.46
	Involved location	Major salivary glands	13	59.1
		Minor salivary glands	9	40.9
	Microscopic differentiation grade	Low	12	54.54
		Moderate	5	22.73
		High	5	22.73

PA: Pleomorphic adenoma, MED: Mucoepidermoid carcinoma

**Figure 1:**
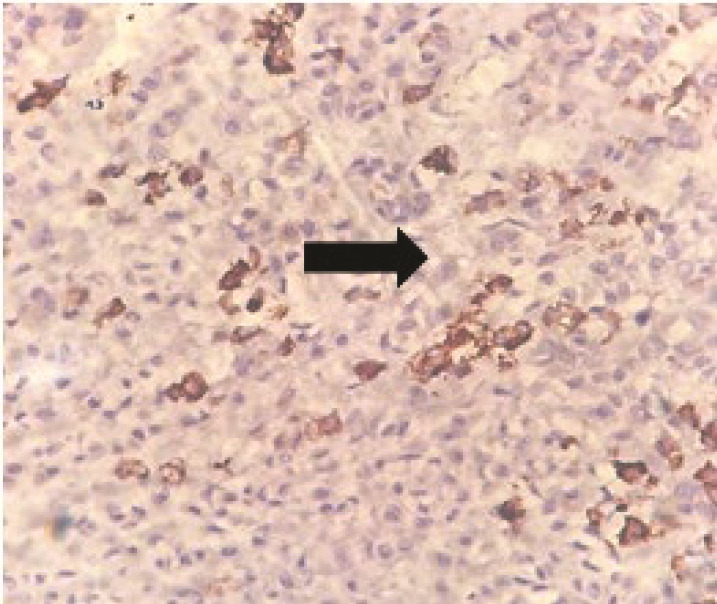
Stained nuclei of pleomorphic adenoma cells (400×)

**Figure 2:**
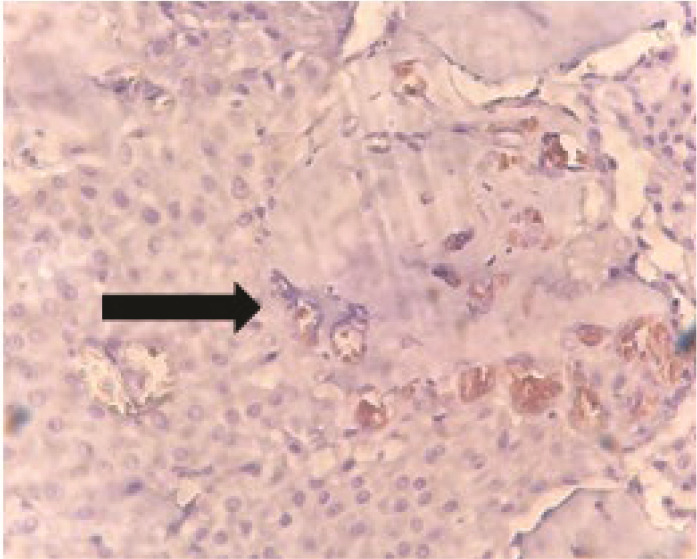
Stained nuclei of mucoepidermoid carcinoma cells (400×)

Of the 36 PA samples, the PR receptor expression level was moderate in only one case, and the rest were negative or had weak expression (Figure 1). PR receptor was weakly expressed in only one sample of MEC and was not expressed in the rest of the cases (Figure 2). 

Most of MEC samples in all microscopic grades had an expression of less than 5% (low grade: 91/6%, moderate grade: 100%, high grade: 100%) and only one low -grade sample had an expression between 5 and 20%. 

The comparison of the expression level of the receptor between two tumors was not statistically significant (*p*= 0.719) (Table 2). Statistical analyses suggested that there was no significant relationship between the level of PR receptor expression in PA and MEC based on background variables (Tables 3-4).

**Table 2:** Comparison of progesterone receptor expression between mucoepidermoid carcinoma and pleomorphic adenoma

Progesterone expression level	Negative <5%	Weak 5-20%	Moderate 21-50%	Strong >50%	*p* Value
Groups					
MEC	21 (95.5%)	1 (4.5%)	-----	-----	0.719
PA	33 (91.7%)	2 (5.6%)	1(2.7%)	-----	

Fisher Exact Test, PA: Pleomorphic adenoma, MEC: Mucoepidermoid carcinoma

**Table 3:** Progesterone receptor expression level in pleomorphic adenoma in terms of background variables

Progesterone expression level			Negative	Positive			*p* Value
Group			<5%	Weak 5-20%	Moderate 21-50%	>50%	
PA	Age	<40	13 (92.9%)	1 (7.1%)	-----	-----	0.689
		≥40	20 (90.9%)	1 (4.55%)	1 (4.55%)	-----	
	Gender	Male	14 (100%)	-----	-----	-----	0.353
		Female	19 (86.36%)	2 (9.09%)	1 (4.55%)	-----	
	Location involved	Major salivary glands	26 (96.29%)	1 (3.71%)	-----	-----	0.148
		Minor salivary glands	7 (77.77%)	1 (11.11%)	1 (11.11%)	-----	

Fisher Exact Test, PA: Pleomorphic adenoma

**Table 4:** Progesterone receptor expression level in mucoepidermoid carcinoma in terms of background variables

Progesterone expression level			Negative	Positive			*p* Value
Group			<5%	Weak 5-20%	Moderate 21-50%	>50%	
MEC	Age	<40	9(100%)	-----	-----	-----	0.394
		≥40	12(92.3%)	1(7.7%)	-----	-----	
	Gender	Male	11(91.7%)	1(8.3%)	-----	-----	0.350
		Female	10(100%)	-----	-----	-----	
	Location involved	Major salivary glands	12(92.3%)	1(7.7%)	-----	-----	0.394
		Minor salivary glands	9(100%)	-----	-----	-----	
	Microscopic grade	Low	11(91.7%)	1(8.3%)	-----	-----	0.646
		Moderate	5(100%)	-----	-----	-----	
		High	5(100%)	-----	-----	-----	

Fisher Exact Test, MEC: Mucoepidermoid carcinoma

## Discussion

PA accounts for 45-75% of all salivary gland tumors and 70-80% of benign salivary tumors [ 22 - 23 ]. Among malignant salivary gland tumors, MEC is the most common tumor constituting 35% of all salivary gland tumors [ 24 ]. These tumors are usually more prevalent in females than males; yet, this sex proportion is different in different tumors and can suggest the role of sex hormones in the histogenesis of salivary gland tumors [ 13 ]. Receptors of steroid hormones such as PR are intracellular proteins that bind to DNA and play the role of regulating cell growth and development. The binding of the hormone to the receptor leads to changes in the morphology of the receptor, followed by the transfer of the receptor- hormone complex to the nucleus [ 8 ]. In the nucleus, this complex binds to specific sequences of nucleotides, leading to transcriptional regulation of genes related to growth factors, degrading enzymes, and components of the extracellular matrix. In this way, tumors that secrete estrogen and PR respond better to hormone therapy compared to tumors without receptors [ 8 ]. The results of the present study were consistent with some studies while contradicted with some others. In Kolude *et al*. study [ 20 ], estrogen was expressed in 6.7% of benign tumors and 28% of malignant tumors. Among the malignant tumors in which estrogen receptor was expressed, 66.7% had high grade and 20% had low grade. PR receptor was expressed only in two cases of adenoid cystic carcinoma and one case of myoepithelioma, and it was not expressed in any of the samples of MEC and PA. This study showed that estrogen receptor expression in high-grade malignant tumors is higher than low-grade tumors and benign tumors. In our study, as in this study, PR receptor was not significantly expressed in salivary tumors, and no significant relationship was observed between PR receptor expression, age, and gender variables. In Nasser *et al*. study [ 13 ], similar to our study, out of 10 MEC samples and 10 PA samples, PR receptor was moderately expressed in only one MEC sample. The present study was consistent with Dori *et al*. study [ 25 ], wherein the PR receptor was expressed in only one sample out of 27 malignant salivary tumor samples and the researchers concluded that hormone therapy exerted no effect on the treatment of malignant salivary tumor and its tumorigenesis; this is consistent with the results of the present study. The present study was consistent with the studies by Seifi *et al*. [ 8 ], Aquino *et al*. [ 11 ], Hsieh *et al*. [ 26 ], and Ito *et al*. [ 27 ] wherein the researchers concluded that there was no significant relationship between the level of PR receptor expression and salivary gland tumorigenesis. In Barrera *et al*. study [ 28 ], contrary to the present study, the expression of estrogen and PR hormone receptors was significant in malignant salivary tumors. On the other hand, in Jeannon *et al*. study [ 29 ], 20% of the samples expressed the PR receptor, while in our study; only 7% of the samples expressed the above receptor. Moreover, in Shick *et al*. study [ 18 ], estrogen receptor was not expressed in any of the adenoid cystic carcinoma samples; yet, PR receptor was expressed significantly. In this study, salivary tumors with more aggressive behavior, such as having a solid histopathological pattern, were expressed with PR receptor; this may indicate a direct relationship between tumor aggressive behavior and PR receptor expression. This study concluded that PR receptor, unlike estrogen, plays a role in adenoid cystic carcinoma tumorigenesis. In Ozono *et al*. study [ 19 ], 80% of the examined malignant tumors (adenoid cystic carcinoma) expressed the PR receptor; the results of our study were contrary to their results. These contradictory results in the expression levels of the receptor may be due to differences in the antibodies used, the criteria used to determine positivity and negativity, and the insufficiency of available samples. Other factors attributed to the different results probably include age, sex, histopathological appearance, and anatomical location of the tumor. The method of performing the immunohistochemical technique, the duration of fixing the paraffinized blocks, staining methods, and study methods (IHC and PCR) affect the results [ 6 ]. In Jeannon *et al*. study [ 29 ], the thickness of the sections, the pH of the buffer used, and the duration of microwave exposure were different from our study. In Nasser *et al*. study [ 13 ], the type of antibody that was different from that in our study can be one of the possible reasons for the disparity in results. The difference in the grading used between our study and Barrera *et al*. study [ 28 ] can cause a difference in the interpretation of the results. In the present study, if less than 5% of tumoral cell nuclei reacted with PR receptor, it was considered negative, which was in agreement with the studies by Tabatabaei *et al*. [ 19 ], Seifi *et al*. [ 8 ], and Nasser *et al*. [ 13 ]; nonetheless, it was different from Ozono *et al*. method [ 19 ] wherein the non-stainability of the nucleus of tumoral cells with PR receptor was considered as negative. In Souza *et al*. study [ 2 ], as in our study, the expression of PR receptor in PA was not significant. In the procedure of conducting this study, if less than 10% of tumoral cell nuclei reacted with PR receptor, they were considered as negative. Nevertheless, in our study, the reaction of less than 5% of tumoral cell nuclei with PR receptor was considered as negative. The reasons for negativity of PR receptor in PA and MEC can be explained as follows.

Development of cancer is a complex process, in the creation of which various factors play a role and a number of markers may be lost during the tumor genesis of cells. PR receptor density and concentration may be low and an adequate response may not be given. Variable criteria in the interpretation of immunohistochemical results can also be one of the reasons for contradictory results obtained from immunohistochemical studies on PR receptor in salivary gland tumors. Other investigations also revealed that estrogen and PR receptors are sensitive to proteolytic enzymes and heat, so the lack of staining and negative response can be caused by the destruction of these receptors during laboratory procedures [ 8 ].

Still, the results of our study, like some previous studies , do not support the role of this hormone in salivary gland tumorigenesis. It should be noted that in addition to the immunohistochemical technique, other methods such as PCR and in situ hybridization can provide more accurate results [ 8 ]. Shick *et al*. [ 18 ] and Ozono *et al*. [ 19 ], who, contrary to our study, found a high expression of PR receptor in salivary tumors, believe that the expression of PR receptor is considered as a good indicator in the prognosis of tumors that are under hormonal treatment. Some researchers believe that the expression of PR is a better marker for responding to hormone therapy compared to estrogen, while in the recent study and Kolude *et al*. [ 20 ], the level of PR expression was not high, and it is not possible to establish a relationship between the level of expression of the marker and microscopic types and tumor progression. 

## Conclusion

PR probably does not play a direct role in tumorigenesis of benign and malignant salivary gland tumors. Based on this, it appears the treatment with PR antagonists, as used in the case of malignant breast tumors, probably cannot be considered as a recommended and common treatment in salivary tumors.
